# Multipurpose Modular Wireless Sensor for Remote Monitoring and IoT Applications

**DOI:** 10.3390/s24041277

**Published:** 2024-02-17

**Authors:** Víctor Sámano-Ortega, Omar Arzate-Rivas, Juan Martínez-Nolasco, Juan Aguilera-Álvarez, Coral Martínez-Nolasco, Mauro Santoyo-Mora

**Affiliations:** 1 Doctorado en Ciencias de la Ingeniería, Tecnológico Nacional de México/IT de Celaya, Antonio García Cubas 600, 38010 Celaya, Mexico; victor.samano@itcelaya.edu.mx (V.S.-O.); juan.aguilera@itcelaya.edu.mx (J.A.-Á.); 2 Maestría en Ciencias en Ingeniería Mecatrónica, Tecnológico Nacional de México/IT de Celaya, Antonio García Cubas 600, 38010 Celaya, Mexico; m2203040@itcelaya.edu.mx; 3 Departamento de Ingeniería Mecatrónica, Tecnológico Nacional de México/IT de Celaya, Antonio García Cubas 600, 38010 Celaya, Mexico; coral.martinez@itcelaya.edu.mx (C.M.-N.); mauro.santoyo@itcelaya.edu.mx (M.S.-M.)

**Keywords:** Internet of Things, remote monitoring, Wireless Sensor Node, Wireless Sensor Network

## Abstract

Today, maintaining an Internet connection is indispensable; as an example, we can refer to IoT applications that can be found in fields such as environmental monitoring, smart manufacturing, healthcare, smart buildings, smart homes, transportation, energy, and others. The critical elements in IoT applications are both the Wireless Sensor Nodes (WSn) and the Wireless Sensor Networks. It is essential to state that designing an application demands a particular design of a WSn, which represents an important time consumption during the process. In line with this observation, our work describes the development of a modular WSn (MWSn) built with digital processing, wireless communication, and power supply subsystems. Then, we reduce the WSn-implementing process into the design of its modular sensing subsystem. This would allow the development and launching processes of IoT applications across different fields to become faster and easier. Our proposal presents a versatile communication between the sensing modules and the MWSn using one- or two-wired communication protocols, such as I^2^C. To validate the efficiency and versatility of our proposal, we present two IoT-based remote monitoring applications.

## 1. Introduction

Nowadays, the Internet is omnipresent almost all over the world and directly impacting on our lives [1]. Consequently, we have reached a new age where humans and the “Things” have become dependent on the Internet to share information constantly. A “Thing” is an entity such as a human being, an animal, a device, or any other element around the globe. Under the context of IoT, these “Things” must be accessible, reconfigurable, reusable, and locatable, and have a unique identity and be remotely controllable [2]. However, these devices must be able to access information or communicate through an Internet connection to allow a continuous information interchange. As part of the IoT, the “Things” are transferred from the physical world to a virtual world, enabling remote accessibility simultaneously. This accessibility has empowered the IoT to be applied in various fields, including environmental monitoring, smart manufacturing, healthcare, smart buildings, smart homes, transportation, energy management, and others [3]. In the literature, we can find some examples related to these fields: in [4], the authors introduce the conversion of a refrigerator into a smart appliance, where the refrigerator is configured to send information, receive continuous updates, and maintain monitoring in its use; also, we can find a voice-activated smart system implemented with IoT, which connects an ESP8266 board with a Google Assistant and a database in Firebase [5]; finally, in [6], the authors propose the management of a smart home with an application developed in Blink App (available for Android and iOS) and linked with an ESP8266 board, which is responsible for sensor data recording and the enabling of actuators associated with programmed tasks in the application.

The reader will find from Section 1.1, Section 1.2 and Section 1.3 the description of our contribution to the topics of Wireless Sensors and Wireless Sensor Networks in the context of engineering applications for IoT. First, Section 1.1 introduces the role of both Wireless Sensors and Wireless Sensor Networks in IoT applications. Then, Section 1.2 concentrates on 13 studies related to Wireless Sensors, where we analyzed the relevant characteristics from their applications. Finally, in Section 1.3, we describe the proposal developed to give our contribution to these fields.

### 1.1. Wireless Sensor and Wireless Sensor Network Role in IoT

**Figure 1:**
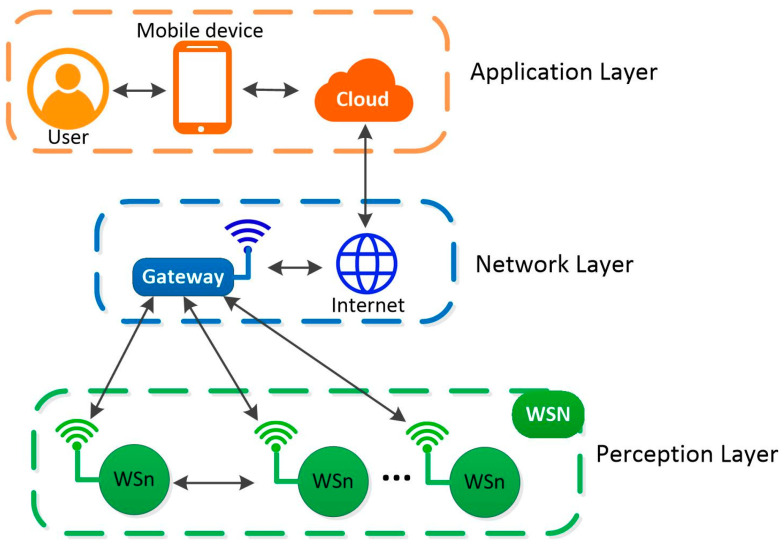
WSN-based IoT architecture.

As stated in [7], the architecture of an IoT includes five elements: elements’ identification, interconnected devices, communication device–device and/or device–application, storing and a data analysis, and data visualizing. The devices connected to the network include sensors, actuators, gateways, or any type of entity contributing to registering, transmitting, and/or processing data. Now, the architecture of an IoT application based on Wireless Sensors is composed of three layers [8,9,10,11,12], the Perception Layer (PL), the Network Layer (NL), and the Application Layer (AL), as can be seen in Figure 1. The first layer consists of Wireless Sensor Nodes (WSn) reading information from their work environment. Later, this information is shared through a common wireless network named a Wireless Sensor Network (WSN). The NL is responsible for holding the routing devices and the Internet network to transmit the collected information from the PL to the Cloud. Finally, the AL organizes, analyzes, and displays the recorded data through Cloud services. Here, the data become comprehensible for the end-user.

**Figure 2:**
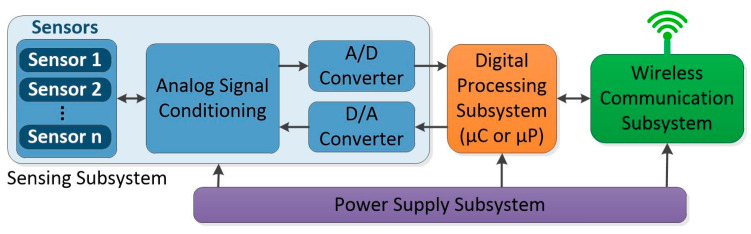
General structure of a WSn.

A Wireless Sensor Node (WSn) is an electronic device integrating a set of transducers to measure physical variables in a working environment. After reading these variables, the data could be transmitted wirelessly to another node, including the central node called the Base Station, or be analyzed by a digital processing unit connected to a node [13]. In general, a WSn is formed by four elements [13,14,15,16]: the sensing subsystem (SS), the Digital Processing Subsystem (DPS), the Wireless Communication Subsystem (WCS), and the power supply subsystem (PSS). This group of subsystems arranged as a WSn is shown in Figure 2. The SS is a set of analogic elements (transducers, filters, amplifiers, comparators, etc.) reading information from physical phenomena to be represented in a digital format with the aid of analog-to-digital converters (ADCs) and reversed to an analogic format with the aid of digital-to-analog converters (DACs). Later, all the recorded information in this subsystem is transferred to the DPS. Once the DPS acquires the information from the SS, it is organized in data packages and sent to the WCS, where a node-to-node or node-to-Base-Station wireless communication channel is established. This wireless communication channel is set by using a short-range radio transceiver; generally, the WCS is responsible for the highest energy consumption in the WSn. Finally, the PSS provides energy for all the subsystems connected to the WSn.

Recently, Wireless Sensors have increased their popularity in the creation of innovative monitoring systems. This results from their capability to access real-time data remotely using Internet networks. However, this capability depends entirely on the network’s bandwidth [14]. In line with this tendency, the increasing number of applications connecting humans and “Things” to the Internet expose several examples of the use of Wireless Sensors with IoT, including smart homes, smart gardening, smart hospitals, smart transportation, smart parking, smartphone detection, smart lighting systems, waste management, water management, and smart product management, among others [17]. Due to the need for an end-to-end communication channel between the devices in a network and a gateway, wireless communication networks play a key role in IoT. These networks can be developed under different wireless protocols, whose election depends on the application requirements such as the range, bandwidth, and energy consumption. The most used technologies in IoT include Wi-Fi, Narrowband IoT, ZigBee, LoRa, Sigfox, LTEM, and low-energy Bluetooth [18]. Another technology widely used for wireless information interchange is the radio frequency identification (RFID). In [19], a system for food quality assessment using RFID is developed. This system includes automated learning to prevent adulterated food consumption. In [20], a measurement platform based on Arduino Nano V3 is presented, which uses an RFID BAP to communicate temperature measurements. The work shows progress in the development of lightweight Wireless Sensors but a connection to the Cloud is not demonstrated. In [21], a passive RFID sensor that is suitable for monitoring different environmental parameters in the agricultural growth process is proposed. It shows that it can measure four variables, temperature, humidity, light, and carbon dioxide, and presents several advantages such as a low cost, a fast response, good repeatability, and stability. It does not have connectivity with the Cloud.

Remote monitoring allows the end-user to use IoT devices to access information through the Internet, to be analyzed and/or displayed with an application. A well-developed application in IoT must include a good selection and use of hardware and software. As a result, the developer will have effective remote monitoring as well as efficient energy management [22]. In addition, some advantages in the use of IoT have been identified, like the continuous sharing of data between the “Things” and the user; this data sharing has improved thanks to Internet accessibility and the advances in specialized devices to facilitate its transferring [7]. As an example, it is worth mentioning the work presented in [23], where the authors placed Wireless Sensors to record the relevant data of a river’s behavior in a server. An application connects directly to this server and presents a comprehensible user interface to visualize the data read by the sensors in quasi-real time. The user can access this information on an LCD connected to a Receiver node, with a PC connected to the Internet, or even with a phone connected to the Internet. It is well known that sensors are employed to register and provide real-time information related to a process. Their implementation contributes to reducing costs and energy consumption and developing smart management systems to enhance their general capacity. For example, we can refer to manufacturing and energy transmission and distribution systems [24].

### 1.2. Wireless Sensors and IoT Applications

Here is a set of published works in different fields from 2018 to 2023 related to Wireless Sensors and IoT.

The first case analyzed conducted the implementation of an architecture for a multi-node measuring system to track, manage, and control energetic resources. In this architecture, each node represents an agent endowed with sensors to measure the variables of interest. All the recorded data of the agents were sent to a master agent, which also counts with a Wi-Fi connection. This proposal was built on a Raspberry Pi, implementing a Modbus RTU communication protocol [24].

In [25], a microcontroller is employed to monitor the provided energy by photovoltaic panels, which are used to recharge batteries. The destination of the recorded data was a Cloud server. A Raspberry fed this server after the microcontroller transferred the data of the measurements. The data on the server contained the current, voltage, and power measurements; these data were visible to any user with permission. By accessing these variables, the user poses valuable information to achieve smart energy management, an early diagnosis of electrical problems, and an enhancement of security, and to perform informed decision making in the use of electrical devices.

The field of microgrids (µG) has been benefited with monitoring systems to diminish the energy consumption from the utility grid and increase the use of renewable energy sources. This is shown in [26] with a system comprising voltage, passive infrared, and photo-resistive sensors, as well as relays to connect/disconnect loads. This system is controlled with a microcontroller, where the loads are switched depending on the consumer demands. All the data from the system are uploaded to the Internet for monitoring.

Similarly, Ref. [27] presents a smart IoT-based Energy Management System built on an architecture that includes multiple nodes focused on monitoring and control functions, a node as the Energy Management System (EMS), and a gateway. The EMS node decides either to turn on or off the connected loads on each of the other nodes; the decisions made by the EMS during time-lapses demanding greater energy consumption are based on the received information from the monitoring process and the priority defined by the user. In this system, the modules communicate node-to-node with a XBee network, and each module is equipped with an acquisition unit to measure the RMS voltage and RMS current. In addition, the active power, apparent power, energy, and power factor are calculated.

The authors of [28] report a system to monitor the energy consumption for household appliances, where the proposal achieves an absolute error lower than 2.5%. The system comprises a Wireless Sensor, a server on ThingSpeak, and an Android application. First, the sensor registers both the RMS voltage and current to compute the instantaneous power and efficiency. Then, the server computes the energy consumption by the appliance and the costs. Finally, the user visualizes the energy consumption and cost in the application. The application also includes a feature to define a billing date and the energy cost.

The proposal presented in [29] offers an approach to create a module capable of connecting to various sensors communicating through analog ports, digital ports, or the I^2^C protocol. If in range, this module can connect to a LoRaWAN zone. After integrating a photovoltaic panel, the system showed an autonomous functioning for long periods. Even though this proposal does not mention using an application to display the recorded data from the sensors, the data are processed in a monitoring center. On the other hand, Ref. [30] presents an internal temperature control in a system that is also based on LoRaWAN. However, the system operation requires different development boards to connect the sensors; meanwhile, the gateway was set in a Raspberry Pi, and the data management was implemented in SQL.

The authors of [31] describe a smart monitoring system consisting of two parts: a “Sender” responsible for sending information to a mobile application developed in an Android and for requesting information or even enabling actuators connected to the “Receiver”. The “Receiver” reads the registered data from a set of sensors and activates/deactivates the actuators as the “Sender” requested. It is possible to identify two limitations in this work; the first is that there is not an Internet connection, which might impact the accessibility to data. The second is the characteristic low range of the LoRa networks.

As we have seen in previous works, there is an extended use of different processing units, including the Raspberry Pi and some microcontrollers. Qatar University developed an energy monitoring system using Raspberry Pi 2 and an XBee-ZB in line with this tendency. This system sends the information to Emncons, which is a data manager in the Cloud specialized in storing and processing data for a later visualization [32]. Similarly, Ref. [33] uses an ESP8266 to develop an open-source smart meter to concentrate a variety of commercial sensors. The application was developed as a smart meter that displays the measurements stored in the Cloud and local measurements of the meter.

The field of agriculture has been identified as an area of opportunity for Wireless Sensors and IoT, too. To manage energy and resources efficiently and to reduce the time invested in work by the farmers, a sensing system was developed whose measurements would be sent to a server and displayed in an application later in an easy-to-read format. The uploading of information was executed by a gateway configured in Raspberry Pi 3. The sensors in the system are connected as nodes and configured with the aid of KIANI (developer: SIXFAB). The setting of the sensors was designed with the humidity and temperature sensors placed on the main board. Meanwhile, the soil humidity sensor was external to the board [34].

The work presented in [35] designed a sensor for healthcare. In this case, the sensor wirelessly registers lumbopelvic kinematics measurements by placing accelerometers and gyroscopes on key locations in the lumbopelvic area. To accomplish the wireless data transmission, the system used Bluetooth technology to send the information to MATLAB for its analysis.

Likewise, a portable system for pulmonary health monitoring was developed in [36]. The monitoring is performed with a piezoelectric sound sensor and an electrocardiogram (ECG) electrode. First, the biopotential and acoustic measurements pass through a 16-channel differential amplifier and a 16-bit ADC; then, the result of this process is transmitted to a computer where the data are processed with MATLAB.

Table 1 summarizes the main characteristics of the WSn presented in all the applications previously described. The information contained in each column, in order, is the consulted reference, the research field of the application, the sensors conforming the WSn, the sensor–DPS communication protocol used, the µC or µP used as DPS, the wireless chip used by the WSn, and the power source of the WSn. It is important to point out that different groups of columns refer to the WSn subsystems, i.e., the third and fourth columns correspond to the SS, the fifth column is the DPS, the sixth column is the WCS, and the seventh column is the PSS.

### 1.3. Our Proposal

As seen in Table 1, to deploy a WSN-based IoT application, it is indispensable to design a set of specific subsystems for each WSn. This represents an increase in and continuous adaptation of the required resources as a new Wireless Sensor for a new application is created. The main objective of our proposal is to develop a multipurpose Modular Wireless Sensor Node (MWSn) for remote monitoring and IoT applications. It is worth noting that our module embeds the DPS and the WCS and presents alternative energy sources for the PSS. Additionally, depending on the application of the MWSn, the user will be able to create and interchange the SS and the PSS modules on demand. This feature makes the MWSn become reconfigurable and versatile with applications in diverse study fields. The design of the MWSn was considered robust to adapt the module to different purposes, and a Mi-Fi modem was added for environments without a Wi-Fi network. In this way, our designed MWSn takes account of the PL and the NL.

We considered a ModeMCU ESP8266 (ESP) development board to design our MWSn. Compared with similar size open-access microcontrollers (e.g., Arduino Nano and Arduino Uno), the node MCU has more powerful resources, is lighter, and includes wireless connectivity [37]. The MWSn is assembled in a protective PLA case and has the following characteristics: a selectable AC/DC source, an I^2^C port, a general-purpose two-bit I/O digital port, a USB port to connect a Mi-Fi modem, a user interface (UI) with two buttons and an OLED screen, an external antenna, a fan cooling system (implemented with an LM35 sensor, a small DC fan, and an internal control system). We strongly believe that this proposal contributes to the topics of Wireless Sensors and Wireless Sensor Networks for engineering applications as listed below:

- It details the design of a multipurpose modular Wireless Sensor for remote monitoring and IoT applications in a way that could be easily reproducible.
- The MWSn is thought to deploy remote monitoring and IoT applications faster and more efficiently, i.e., the developer will focus only on the design and development of the SS and/or the PSS.
- The physical variables measured by the MWSn do not depend on the complete module but on the sensor module designed for a specific purpose. This characteristic enhances the versatility and reconfigurability of the module.
- The MWSn includes an I^2^C bus with a capacity for up to 128 devices and a general-purpose two-bit I/O digital port to create a one- or two-wired communication channel to configure protocols such as 1-Wire, RS232, RS485, UART, and Modbus RTU. With these two ports, we extend the type of external modules that can be connected to the MWSn.
- In existing works like [29,31,33], WSn are presented, mentioning that they can be used in different application fields. However, they design the entire WSn specifically, so its use in a different application would require redesign of the entire device. With the MWSn presented in this research, this redesign is only required in the SS, so our proposal has advantages compared to similar existing works.
- An internal DC power supply is configured to be energized by either an AC source or an external DC source module. For example, the DC source could be a battery, a photovoltaic system, or any energy harvester.
- It includes a USB port configured to supply energy to a Mi-Fi modem. This modem allows the MWSn to establish an Internet connection by a GSM network to support applications that do not count with a Wi-Fi network.
- While the MWSn was initially designed as a sensing module, its functionality is not restricted to following only this purpose. Therefore, it is possible to create modules comprising actuators to maintain communication with the MWSn.

This article is organized as follows: Section 2 describes the MWSn and its implementation for remote monitoring applications. Section 3 shows the MWSn prototype and two experiments that were performed to validate our design. Finally, Section 4 contains our conclusions and the next steps in our work.

## 2. Proposed MWSn for Remote Monitoring and IoT Applications

**Figure 3:**
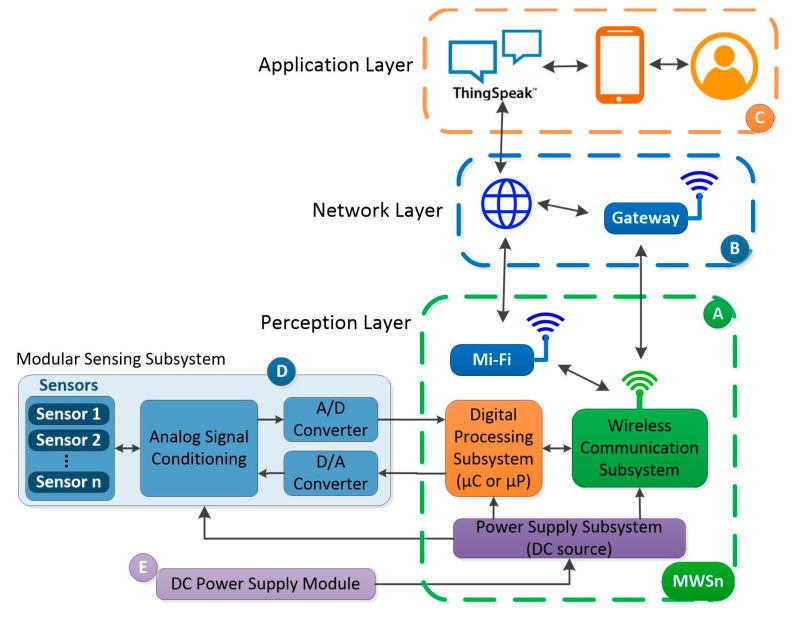
General structure of the MWSn-based IoT application.

The main goal of the MWSn is to deploy WSN-based IoT applications faster and more easily. For this reason, the MWSn was validated with a remote monitoring application. Figure 3 shows the structure of the implemented monitoring application in this work, which consists of five blocks (A to E). Block A refers to the MWSn designed in our work and, at the same time, refers to the PL. Blocks B and C correspond to the NL and AL, respectively. A sensing module corresponding to the SS in the MWSn is represented by Block D. Finally, Block E consists of a power supply module, i.e., the PSS. These blocks are described extensively in the following sections.

### 2.1. MWSn Description (Block A)

**Figure 4:**
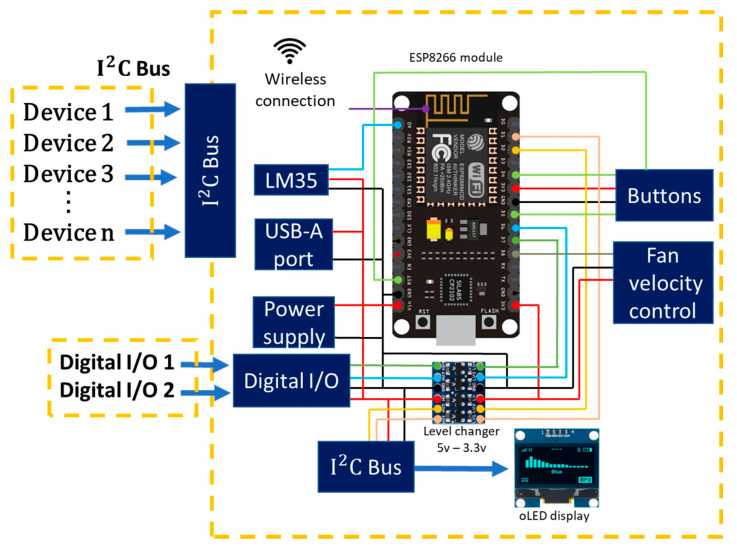
MWSn’s architecture.

The MWSn’s architecture is shown in Figure 4. In this architecture, the DPS and WCS are embedded into an ESP development board, the PSS consists of a DC power supply, and there are ports to connect external modules to operate as the SS and, alternatively, as the PSS. Therefore, developing a WSn for a specific application is reduced to the SS designing process.

As the ESP works with logic levels ranging from 0 to 3.3 V, it was necessary to adapt a level changer to accomplish a satisfactory coupling with TTL-level signals. One of the advantages of using this board is that it embeds a Wi-Fi module capable of connecting to 2.4 GHz networks. This allows the establishment of communication with various APIs, data managers in the Cloud, and other devices that also count with a protocol based on Wi-Fi. In addition, the ESP is an open-access technology.

To enhance the quality of the Wi-Fi connectivity, the MWSn is adapted with an external antenna. The MWSn circuit is protected with a casing; however, this will impact the quality of the signal perceived by the ESP. The added antenna is not part of the original ESP hardware, so it had to be modified by adding a surface mount IPEX connector to the ESP on-board antenna. Additionally, the design adds a USB-A port to energize a Mi-Fi modem to access a GSM network as an alternate Internet connection. This feature represents an advantage because some locations do not have access to the Internet with a Wi-Fi network. We could take advantage of this feature by monitoring physical variables in greenhouses, fields, distant communities, etc.

It was necessary to add a cooling system due to the heat produced by the electronic components; this system becomes even more necessary if the MWSn is placed in environments with warm weather. The control of the cooling system measures the inner temperature of the MWSn case with an LM35 sensor and activates a PWM-controlled fan when the temperature increases significantly. The ESP is responsible for executing this control for the cooling system.

The interaction between external modules and the MSWn can be established in either of two approaches. The first uses a general-purpose I/O port (Digital I/O in Figure 4) where the communication protocol can be set to be one- or two-wired (e.g., 1-Wire, RS232, RS485, UART, Modbus RTU). The second method is an I^2^C bus, which allows a connection with up to 128 devices. Both communication ports are directly coupled to the level changer to operate with logic levels ranging from 0 to 5 V. Also, these ports supply a 5 V line for the connected devices.

In situ monitoring is available in the MWSn with a UI containing an OLED screen (connected to the I^2^C Hub) and two buttons. The UI provides the user with information read from the connected devices in both the Digital I/O and I^2^C ports and the Internet connection status. There is also a button to reset the MWSn whenever needed.

Both AC and DC sources can supply voltage to the MWSn. The source selection is made with a switch, which defines if an internal DC power source supplies the MSWn or if the MWSn is supplied by an external DC source connected to a jack. This feature allows the integration of a module to act as the PSS and to adapt the MSWn to a specific application, i.e., it will be possible to supply the required voltage depending on the available sources. The DC sources include batteries, photovoltaic panels, and energy harvesters.

Finally, the MWSn has a switch that works as an energy source coupler/decoupler to update the ESP firmware. This prevents unexpected functioning while updating the firmware.

#### 2.1.1. MWSn Firmware

**Figure 5:**
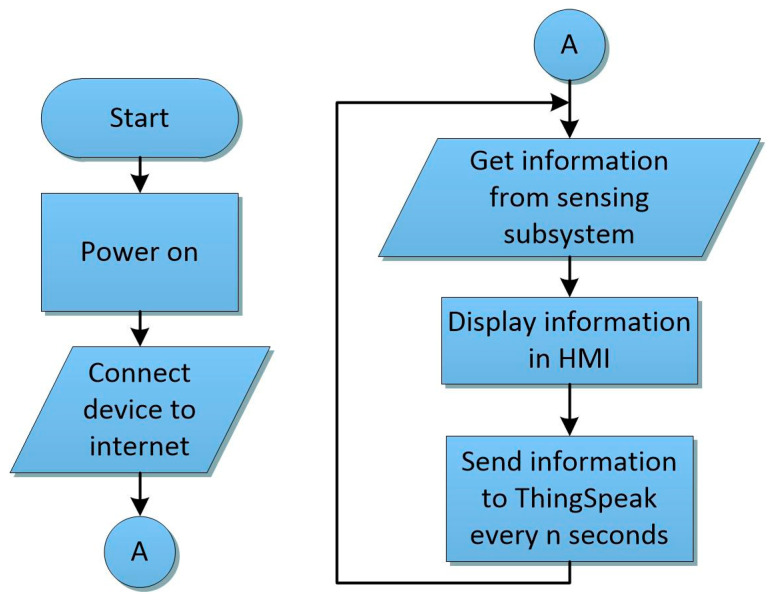
Proposed firmware for the MWSn.

Depending on the peripheral modules connected to the MWSn, specific firmware may be required on the MWSn to establish a correct operation (communication). However, we propose installing general firmware following the process shown in Figure 5. Then, to acquire data from the peripheral connected in the MWSn and to send these data to the Internet, the ESP executes the following steps: firstly, it checks the state of the Wi-Fi connection (the status is visible for the user on the OLED screen), and if there is not an established connection, the ESP tries to reconnect to the network; secondly, the ESP requests data from the peripheral modules connected to any of the available communication ports (i.e., the Digital I/O port or the I^2^C bus) and stores the received information locally; thirdly, the available stored data in the ESP, which can be selected by the user with the navigation buttons, are updated on the OLED screen; fourthly, the recorded data from the peripheral devices are sent wirelessly by the WCS to another node or a server in the Internet. In this work, ThingSpeak was selected as the Cloud server to store the read data from the sensors.

#### 2.1.2. Design of the MWSn Case and Circuit

**Figure 6:**
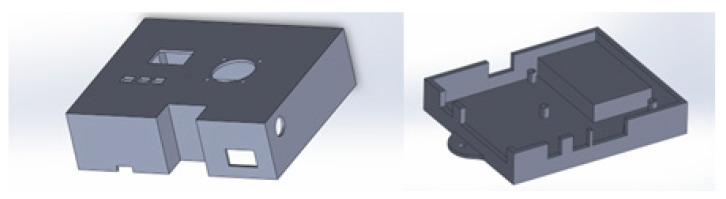
Protective case designed for the MWSn.

A protective case was designed to protect the electronic components and the main board of the MWSn (see Figure 6). Since we were interested in creating an affordable case for low-volume production and availability in a short time, we opted for a fast-prototyping technique. The case was designed using CAD software (SOLIDWORKS® 2018) and printed using PLA. As shown in Figure 6, the design of the case has the necessary holes and spaces to hold the communication ports to connect peripheral modules and a serial port for firmware updates, the voltage supply inputting with its corresponding switch, the electrical fan inputting with its vents, and the elements of the UI (buttons and an OLED screen). To be practical, the protective case also includes a set of holes to be fixed on electric boards or a wall.

**Figure 7:**
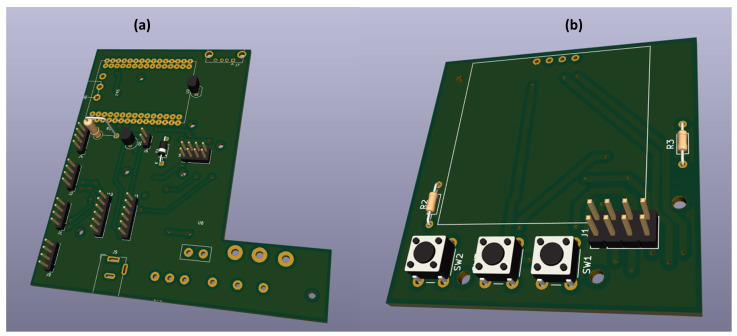
MWSn electronic components: (**a**) bottom PCB model, (**b**) top PCB model.

Similarly, two printed circuit boards (PCBs) were designed with the aid of CAD software; these PCBs can be seen in Figure 7. The first PCB, which we call the bottom PCB and is shown in Figure 7a, was designed to interconnect the ESP with the communication ports through coupling with the logic level changer, the fan power circuits, the internal temperature sensor, and the corresponding connections for the power supply and its selector switch. The second board, called the top PCB, contains the UI of the MWSn, i.e., the navigation buttons and the OLED screen. The top PCB design can be seen in Figure 7b.

### 2.2. Network Layer (Block B)

It is possible to achieve an Internet connection with the MWSn by two alternatives: a Wi-Fi network or a Mi-Fi modem. The Mi-Fi modem requires a SIM card to access the Internet through a GSM network, which allows the MWSn to be implemented in locations that do not have a Wi-Fi network. For instance, we validated the benefit of this feature by monitoring the environmental variables of a greenhouse located in a field. As a result, we present an innovative application of Wireless Sensor Networks in agriculture.

### 2.3. Application Layer (Block C)

The accessibility and visualization of data are part of the objectives of remote monitoring and IoT applications. For this reason, different companies offer services to store, access, and visualize data in the Cloud, from which we consulted ThingSpeak. ThingSpeak offers Cloud databases with the possibility of visualizing the stored data in a chart format. Additionally, ThingSpeak includes in-line data processing applications with MATLAB. As part of the tools available in this in-line processing, the user can compute data statistics and program alerts and timers to execute a task. Due to these features, we decided to use ThingSpeak as part of our monitoring system. ThingSpeak is projected to be a reference tool in developing more complex IoT applications.

### 2.4. Modular Sensing Subsystem (Block D)

It is important to remember that the main goal of this work is to present an alternative for IoT developers to launch their applications faster. Therefore, the MWSn presented here reduces the focus to the design of modular SS while developing a new IoT application. The possibility of including external and modular SS ad hoc to a desired application makes our proposed system versatile and scalable.

Two sensor modules were designed to evaluate the MWSn’s precision in two real applications. As the first case, the MWSn was set up to monitor the energy consumed by a 3D printer during one working cycle, i.e., the printing of a model, from turning on the printer until the printed piece is finished. The second case consisted of monitoring the relative humidity and temperature in a greenhouse.

**Figure 8:**
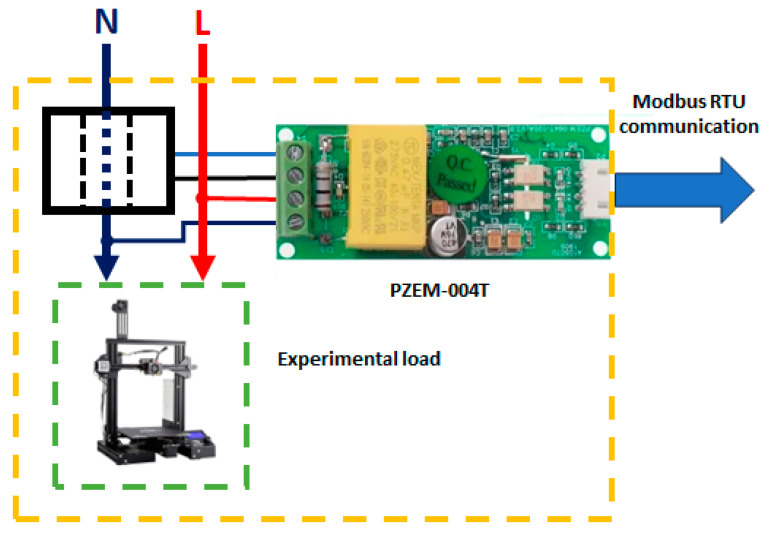
Architecture of the AC sensor module.

In case 1, an AC sensor module (see Figure 8) with a PZEM-004T sensor was developed. This sensor measures loads connected to AC lines, reading six electric variables: the RMS voltage, RMS current, active power, power factor, frequency, and energy. In addition to the characteristics presented in Table 2, the sensor has a Modbus RTU to communicate with other devices.

**Figure 9:**
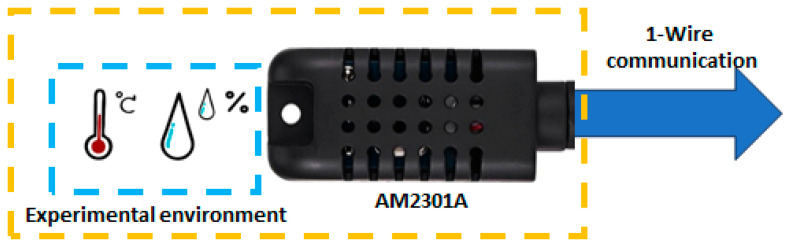
Architecture of the relative humidity and temperature sensor module.

Correspondingly, case 2 considered an application to monitor a greenhouse’s relative humidity and temperature. Thus, we selected an AM2301A sensor, as shown in Figure 9, which is broadly used in temperature control, air conditioning, weather stations, and humidity regulation applications. The sensor requires a 1-Wire communication and has the characteristics listed in Table 3.

### 2.5. DC Power Supply Module (Block E)

The design of the MWSn considered both AC and DC connections for power supply; the selection of the provided source is made with a switch. When the MWSn is connected to AC, the voltage is transferred to an internal AC/DC converter to produce 5 V. On the other hand, when the user selects a DC source, it must be connected to a jack. This feature allows the MWSn to use other types of energy sources in locations without a utility AC grid. Namely, these DC sources could be a battery, a photovoltaic panel, or, in general, an energy harvester. In this study, the MWSn was connected to the utility AC grid to use the internal DC source.

## 3. Results

Here, the results from the development and validation of the MWSn are shown. The section presents the experimental prototype built as MWSn and finishes with the data analysis acquired during the two proposed monitoring applications. In the two experiments, the MWSn was fed with AC through the internal AC/DC converter to produce 5 V.

### 3.1. MWSn Prototype

**Figure 10:**
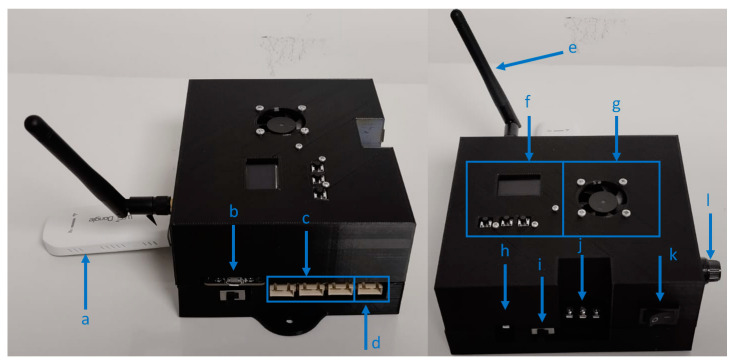
MWSn prototype and its parts.

Figure 10 shows the MWSn prototype built for our research. The elements shown are (a) the Mi-Fi modem connected to the USB-A port, (b) the ESP serial port, (c) a set of connectors for the I^2^C bus, (d) the general-purpose Digital I/O port, (e) the external antenna, (f) the set of buttons and the OLED screen used as UI, (g) the fan used as part of the cooling system, (h) the jack for DC sources, (i) the switch to select the input energy source, (j) the AC input connectors, (k) the on/off switch of the internal DC source contained in the MWSn, and (l) a protection fuse connected to the AC source input.

### 3.2. Energy Remote Monitoring

**Figure 11:**
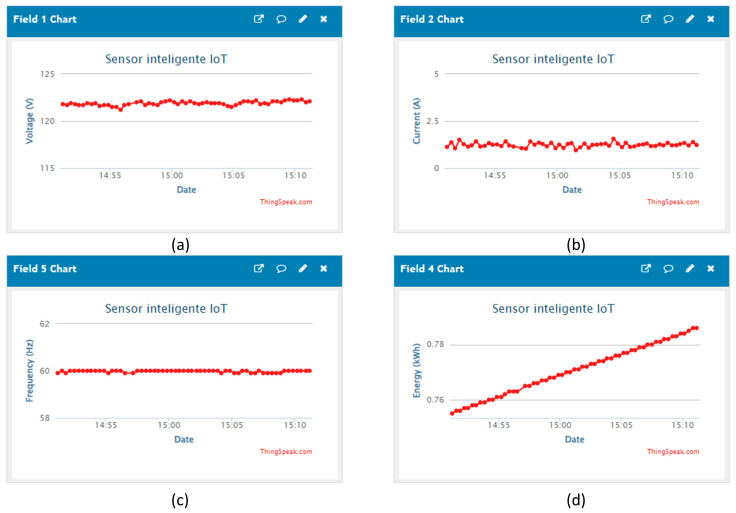
Data sent to ThingSpeak API by the MWSn. Plots are for the following variables: (**a**) voltage, (**b**) current, (**c**) frequency, and (**d**) consumed energy.

This first case of the analysis registered the energy consumption of a 3D printer during a run. The monitoring module recorded the RMS voltage, the RMS current, the active power, the power factor, the frequency, and the energy consumption by the printer. The registered data were sent to ThingSpeak every 20 s for 8 h; the elapsed time corresponds to manufacturing a 3D model. To illustrate the uploaded data to ThingSpeak, Figure 11 shows different plots with the voltage, current, frequency, and energy demanded by the 3D printer.

**Figure 12:**
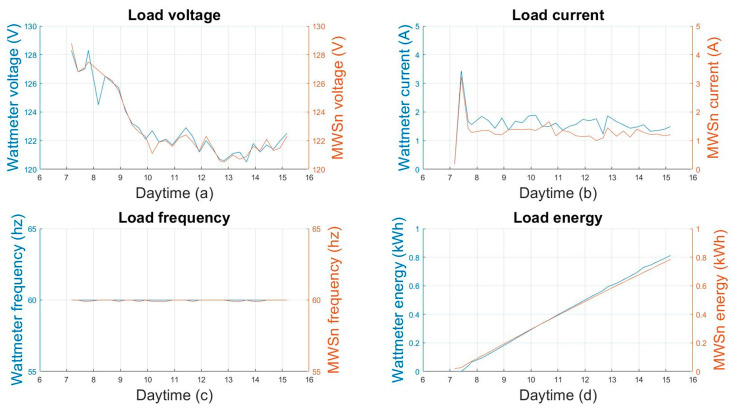
Comparison of the measurements made with the MWSn and wattmeter: (**a**) voltage, (**b**) current, (**c**) frequency, and (**d**) energy consumption.

The measurements made by the AC sensor module were compared with a commercial wattmeter Steren HER-432. This wattmeter measures currents in the range of 1 mA to 15 A. However, the manufacturer does not provide information on its accuracy and resolution nor on the rest of the variables it measures. Every 15 min, a measure was taken using the commercial wattmeter during the 8 h of the test. A visual comparison of the behavior of both measurements is shown in Figure 12, where the blue line corresponds to the measurement made with the wattmeter, and the orange line corresponds to the measurement made by the MWSn. The absolute error was calculated to know the discrepancy between the MWSn and the wattmeter, taking the measures made with the wattmeter as reference values. The calculated errors for each variable are concentrated in Table 4.

### 3.3. Temperature and Humidity Remote Monitoring

**Figure 13:**
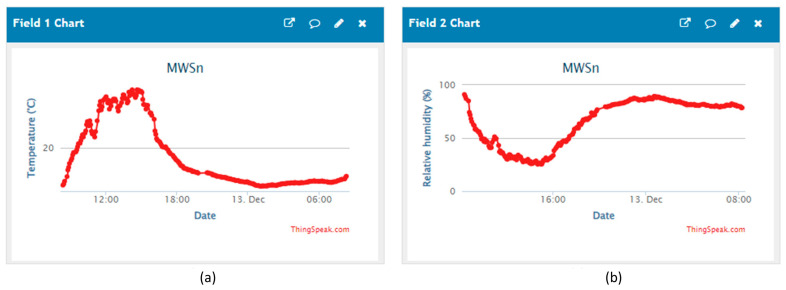
Data sent to ThingSpeak API by the MWSn. Plots: (**a**) temperature and (**b**) relative humidity.

Similar to the previous case, this test required the design of an SS to remotely monitor the relative humidity and temperature in a greenhouse. The registered data by the MWSn were uploaded to ThingSpeak every 5 min for approximately 12 h. A plot with the measurements of relative humidity and temperature registered in ThingSpeak by the MWSn is shown in Figure 13.

**Figure 14:**
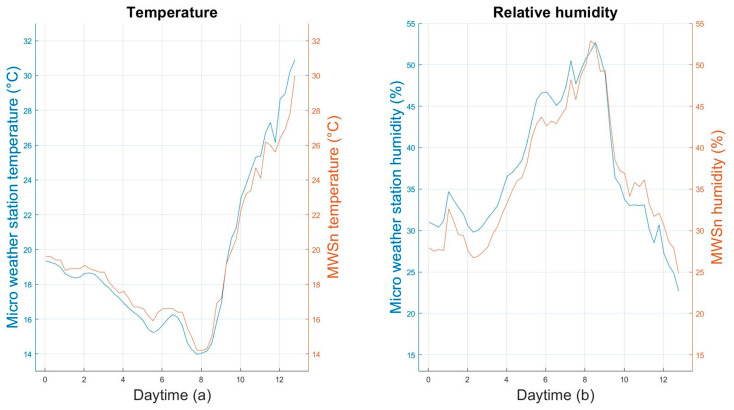
Comparison of the measurements made with the MWSn and the micro-station: (**a**) temperature and (**b**) relative humidity.

The monitoring system developed for this application was compared with a weather micro-station for agriculture HOBO Micro Station H21-USB with sensor S-THB-M008, and the measuring characteristics of this micro-station are shown in Table 5. A register was taken from the micro-station every 15 min during the same 12 h of the MWSn test. The behavior of the registered data from both the MWSn and the micro-station can be seen in the plots of Figure 14. The discrepancy between the MWSn and the micro-station was calculated with the absolute error, where the reference values were the measurements from the micro-station. The results from these calculations are concentrated in Table 6.

## 4. Conclusions and Future Work

It is important to note that the versatility of the MWSn prototype was demonstrated after its implementation in two different applications, each with unique conditions like the use of different communication protocols, sensors, and environmental conditions. Moreover, it was possible to launch each application by only focusing on the design and development of the SS and/or the PSS. This characteristic of the proposed system directly impacts the reduction in time and resources while developing a new remote monitoring IoT application. Commercial systems were used to validate the MWSn functionality; however, the contribution of this work lies in the capability of implementing remote monitoring and IoT applications quickly and easily. The observed measurement differences depend on the sensors selected for the MWSn implementation and not directly on the MWSn described and implemented in this work. More accurate measurements can be achieved by using a more precise sensor, but the accuracy is not directly a characteristic of the MWSn. For this reason, the experimental setup is presented briefly just to exemplify potential uses of the MWSn, and does not include a more exhaustive analysis under various conditions.

The inclusion of different types of communication protocols makes it possible to extend the capability to adapt a diversity of sensing modules. Therefore, the application fields for Wireless Sensors would see faster developments that satisfy their demands. Among the communication protocols the proposed system can handle in this work, we can find I^2^C, 1-Wire, RS232, RS485, UART, and Modbus RTU. Additionally, the possibility to select between two different power sources (AC and DC) allows the user to use the prototype in locations with access to the utility AC grid and/or with DC sources such as a battery or an energy harvester.

The MWSn prototype’s versatility was demonstrated by its application in two different fields and its adaptability to work in different environments and locations with restricted resources. This can be seen in case 2, where the MWSn remotely monitored a greenhouse. Under the environmental conditions of the greenhouse, the MWSn prototype could connect to the ThingSpeak server in the Cloud and maintain a register of the relative humidity and temperature for 12 consecutive hours. The task was accomplished thanks to the inclusion of a Mi-Fi module to create access to the Internet by a GSM network.

Overall, the utility and efficacy of our prototype during a real application were demonstrated after its comparison versus commercial monitoring systems (a wattmeter and a weather micro-station). In [29,31,33], WSn are presented, mentioning that they can be used in different application cases given that the controllers used for the DPS have multiple communication protocols and I/O ports. However, the applications in which they are used are specific, as are the devices designed, so their use in a different application would require redesign of the device. With the MWSn presented in this research, this redesign is only required in the SS, so our proposal has advantages compared to works like those presented in [29,31,33].

Although the MWSn offers wide versatility when it comes to adapting it to different applications, there are certain limitations in this process. These limitations have to do with three key aspects: energy consumption, data loss due to network failures, and the communication protocols used by the sensors to be connected.

Regarding power consumption, although the MWSn can be powered by an external DC source, making it mobile or independent of the electrical power grid (with a battery, for example), the general firmware proposed in Section 2.1.1 does not contemplate the reduction in energy consumption. In general, the WCS is the system that consumes the most energy, so if a battery is used, it is recommended to use the Deep Sleep mode of the ESP, which will drastically reduce the consumption of the WCS. However, if the sensors consume large amounts of energy in the standby state, or if the Mi-Fi modem is being used, it will be necessary to include an element to disconnect it from the power source when they are not being used. As a future improvement, this disconnection element will be integrated into the MWSn.

Regarding data loss due to connection loss, the MWSn does not include local storage. Therefore, if it is implemented in an area with intermittent Internet connection, it is recommended to store the variables in the ESP memory, in case of connection loss, for later sending. An alternative is including an external storage element that could be connected to the I^2^C bus.

Finally, regarding the protocols of the sensors to be connected, it is important to consider that the I2C protocol is short-range and that in addition to this protocol, an additional one- or two-wire protocol can be implemented in the Digital I/O port (e.g., 1-Wire, RS232, RS485, UART, and Modbus RTU). Therefore, if a sensor does not have communication through one of these protocols, it will be necessary to convert its protocol to one of those accepted by the MWSn. As a future improvement, it is proposed to analyze different controllers with the aim of including one, with the possibility of including more communication protocols.

## Figures and Tables

**Table 1:** WSn characteristics extracted from the literature [24,25,26,27,28,29,30,31,32,33,34,35,36].

Ref.	Application	Sensors	Sensing to DPS Communication	µC or µP Used as DPS	Wireless Chip	Power Supply
[24]	Energy	Voltage, Current	modbus RTU	Raspberry Pi 3	BCM43438 (on board)	DC power supply
[25]	Energy	Voltage, Current	Analog inputs	Arduino UNO and Raspberry Pi 3	BCM43438 (on board)	DC power supply
[26]	Energy	Voltage, Current	UART and analog inputs	PIC16F877A	Not mentioned	DC power supply
[27]	Energy	Voltage, Current	Analog inputs	Arduino UNO	Zigbee	DC power supply
[28]	Energy	Voltage, Current	Analog inputs	Arduino UNO	ESP8266	DC power supply
[29]	Multiple	Multiple	ADC, digital, and I^2^C	Not mentioned	LoRa module	Photovoltaic array
[30]	Agriculture	Temperature, Soil moisture	SPI and I^2^C	Raspberry Pi 3, LoPy4	LoRa module	Photovoltaic array
[31]	Multiple	Multiple	Analog inputs, digital inputs, and I^2^C	ESP32	ESP32	DC power supply
[32]	Air quality	Air quality	I^2^C	Raspberry Pi 2	Xbee-ZB	DC power supply
[33]	Multiple	Voltage, Current	Analog inputs	Arduino UNO	ESP8266	DC power supply
[34]	Agriculture	Temperature, Humidity, Soil moisture	Analog inputs	ATmega238P, Raspberry Pi 3	cc1101 Low-Power Sub-1GHz	Battery
[35]	Healthcare	Inertial measurement unit	Analog inputs	ARM Cortex M0	nrf51822	Battery
[36]	Healthcare	Piezoelectric	Analog inputs	ARM Cortex M	nrf 52x BLE5	Battery

**Table 2:** PZEM-004T measuring characteristics.

	Voltage	Current	Active Power	Energy	Frequency	Power Factor
Range	80–260 V	0–100 A	0–23 kW	0–9999.99 kWh	45–65 Hz	0–1
Resolution	0.1 V	0.001 A	0.1 W	1 Wh	0.1 Hz	0.001
Accuracy	0.50%	0.50%	0.50%	0.50%	0.50%	1%
Starting measuring	-	0.2 A	0.4 W	-	-	-

**Table 3:** AM2301A measuring characteristics.

	Temperature	Relative Humidity
Range	−40–80 °C	0–100%
Resolution	0.1 °C	0.1%
Accuracy	<±0.5%	2%

**Table 4:** Absolute errors calculated for the variables of interest in the energy monitoring application.

Absolute Error	Voltage (V)	Current (A)	Active Power (W)	Energy (Wh)	Power Factor	Frequency (Hz)
Mean	0.34	0.28	9.24	0.01	0.07	0.04
Maximum	2.46	0.56	28.73	0.04	0.14	0.1
Minimum	0	0.02	0.3	0	0	0

**Table 5:** Weather micro-station measuring characteristics.

	Temperature	Relative Humidity
Range	−40–75 °C	0–100%
Resolution	0.02 °C	0.1%
Accuracy	±0.21%	±2.5%

**Table 6:** Absolute errors calculated for the variables of interest in the humidity and temperature monitoring application.

Absolute Error	Temperature (°C)	Relative Humidity (%)
Mean	0.64	2.42
Maximum	2.42	4.1
Minimum	0.02	0.3

## Data Availability

Data are contained within the article.
